# Moral foundations theory and the narrative self: towards an improved concept of moral selfhood for the empirical study of morality

**DOI:** 10.1007/s11097-023-09918-x

**Published:** 2023-06-08

**Authors:** Tom Gerardus Constantijn van den Berg, Luigi Dennis Alessandro Corrias

**Affiliations:** 1 Department of Engineering Systems and Services, Faculty of Technology, Policy, and Management, Delft University of Technology, Delft, The Netherlands; 2 Department of Legal Theory and Legal History, Faculty of Law, Vrije Universiteit Amsterdam, Amsterdam, The Netherlands

**Keywords:** Moral values, Moral self, Moral Foundation Theory, Narrative self, Ricoeur

## Abstract

Within the empirical study of moral decision making, people’s morality is often identified by measuring general moral values through a questionnaire, such as the Moral Foundations Questionnaire provided by Moral Foundations Theory (MFT). However, the success of these moral values in predicting people’s behaviour has been disappointing. The general and context-free manner in which such approaches measure moral values and people’s moral identity seems crucial in this respect. Yet, little research has been done into the underlying notion of self. This article aims to fill this gap. Taking a phenomenological approach and focusing on MFT, we examine the concept of moral self that MFT assumes and present an improved concept of moral self for the empirical study of morality. First, we show that MFT adopts an essentialist concept of moral self, consisting of stable moral traits. Then, we argue that such a notion is unable to grasp the dynamical and context sensitive aspects of the moral self. We submit that Ricoeur’s narrative notion of identity, a self that reinterprets itself in every decision situation through self-narrative, is a viable alternative since it is able to incorporate context sensitivity and change, while maintaining a persisting moral identity. Finally, we argue that this narrative concept of moral self implies measuring people’s morality in a more exploratory fashion within a delineated context.

## Introduction

Over the last few decades, the empirical study of moral decision-making has established itself as an important sub-field of psychology, known as moral psychology. Within this field, measuring people’s basic and general moral values, through a general moral questionnaire, has become a common academic practice to map out people’s morality (Curry, et al., 2019; Graham, et al., 2013). Furthermore, these measures have been used to investigate the influence of people’s morality on other attitudes and behaviour (e.g., Clark, et al., 2017; Cohen, 2014; Dickinson, et al., 2016; Graham, et al., 2009; Hoover, et al., 2021; Miles, 2015; Nilsson, et al., 2016; O’Grady, et al., 2019; Qian and Yahara, 2020; Vainio and Mäkiniemi, 2016).

Theories and accompanying questionnaires that are used to measure people’s moral values are, for example, Schwartz Theory of Basic Values (Schwartz, 1992; Schwartz, et al., 2012)^1^ and Moral Foundations Theory (MFT) (Graham, et al., 2013; Haidt & Joseph, 2004). A more recently developed one is Morality As Cooperation theory (MAC) (Curry, et al., 2019). These, what we will call, ‘empirical moral value theories’ define people’s morality through a set of distinct basic moral values. These moral values are regarded as psychological mechanisms that were formed during the evolution of the human being and which are further individually developed during someone’s life. The extent to which an individual has developed a general moral value as part of his or her morality is empirically measured through a generic questionnaire.

Though these values are often presented as determinants of behaviour, their success in predicting people’s actual moral behaviour has been disappointing (Boyd, et al., 2015; Graham, et al., 2012). Studies that have specifically investigated the link between general moral values and specific moral behaviours report weak associations at best (e.g., O’Grady, 2019; Schier, et al., 2016; Van den Berg, et al., 2020; Van den Berg, et al., 2022)^2^. It is far from clear whether people who score higher on a general moral value when filling in a questionnaire also show more behaviour in accordance with that value (Graham, et al., 2012). This is problematic as predicting and explaining individual differences in actual moral behaviour seems to be a central goal when measuring people’s morality (Ellemers, et al., 2019), and as such a central goal of moral psychology.

When considering this issue, one element that catches the eye is the general character of the measured moral values that are attributed to the individual, and the contextless manner in which these are measured (Van den Berg, et al., 2022; Schein, 2020). In light of psychological studies that have emphasized the situational (e.g., Doris and Doris, 2002; Ross and Nisbett, 2011), the social contextual (e.g., Potter and Wetherell, 1987; Southerton, et al., 2004) and issue-contingent nature (Jones, 1991) of (moral) values and behaviour, it is questionable whether regarding such general individual measures as direct determinants of behaviour is in accordance with important presuppositions of how people morally function. This brings us to question some of the more fundamental assumptions underlying general moral value theories. In particular, it raises questions about a concept that seems to be central to our moral functioning and our relationship with moral values, namely that of the moral self. With this, we mean a concept of moral identity that corresponds to the agent of moral decision-making and action. Empirical moral value theories assume a certain conception of moral self, underlying their theory and measurement strategies, as they, in fact, identify people in moral terms. However, what their concept of the moral self entails is not clear. The question then is what this implicit moral self, underlying empirical moral value theories, looks like; how it should be evaluated and, possibly, improved; and what this means for the method of measuring morality’s influence on behaviour.

In this paper, we investigate and critically assess the concept of moral self that underlies empirical moral value theories and argue for an improved concept of moral selfhood for the empirical study of morality. For this purpose, we confine ourselves specifically to Moral Foundations Theory (MFT) and its Moral Foundations Questionnaire (MFQ). We will take the following approach: first, we explicate the implicit notion of the moral self that MFT assumes through a description and analysis of the theory and questionnaire. This results on the one hand in a concept of moral self that consists of stable moral traits, while on the other hand the theory seems to adopt aspects of virtue theory that indicate a more flexible and dynamic moral self, without further developing such a concept. Then, to make sense of this somewhat ambivalent result, we turn to a field that has extensively studied the self: phenomenology. More particularly, we describe and discuss Ricoeur’s concept of narrative moral self (Ricoeur, 1992). This elaboration is used to interpret and evaluate MFT’s concept of moral self and, at the same time, present the narrative moral self as a more viable alternative. In the discussion, we make a few suggestions as to how insights from Ricoeur’s narrative self could possibly enhance the empirical measurement of the influence of moral considerations on behaviour.

## Moral foundations theory and the implied moral self

In our analysis, we focus explicitly on MFT because it is arguably one of the more prominent empirical moral value theories within moral psychology today. Furthermore, it is referred to and used in other fields than moral psychology to study the moral influence on decision-making, such as consumer and environmental studies (e.g., Chowdhury, 2019; de Jonge and van Trijp, 2014; Vainio and Mäkiniemi, 2016) and behaviour during the COVID-19 pandemic (Presti, et al., 2021; Diaz & Cova, 2022). Another reason is that the theory has been extensively described over the last two decades in several empirical and more theoretical papers, which can be used to derive the concept of moral self that it assumes. In the following, we will explicate this implicit conception of the moral self through a description and analysis of the theory and its questionnaire.

### Moral foundations theory

Moral Foundations Theory (MFT) positions itself opposite to rational and monist models of morality, such as the Kohlbergian model of moral development (Kohlberg, 1969, 1984). Instead, MFT claims that our morality consists of a plurality of fundamental moral values^3^, the so-called moral foundations, which intuitively influence our decision-making. Next to moral pluralism and intuitionism, it takes two other elements as central to its theory: nativism and cultural learning. Furthermore, MFT presents itself as a descriptive moral theory, it aims to describe which moral values people actually have, instead of making normative claims about which they should have. People’s moral foundations are measured through the accompanying Moral Foundations Questionnaire (MFQ) (Graham, et al., 2013). We will now briefly go into the four central elements of the theory and, subsequently, into the structure of MFQ.

The first element MFT regards as central to the theory is *pluralism*. It consists in the fact that the theory distinguishes more than one fundamental moral value, i.e., the moral foundations. At this point, at least five moral foundations are distinguished: care/ harm, fairness/ cheating, loyalty/ betrayal, authority/ subversion, and sanctity/ degradation. 4

Secondly, the moral foundations are described as *innate* psychological learning modules or mechanisms that developed as adaptive solutions to distinct social problems of (group) survival, during human’s evolution. As innate learning modules, they are part of every individual’s moral mind in advance of experience. This means they bear a universal *a priori* sensitivity to certain moral beliefs, values, virtues, concepts, principles, judgments etc. congruent to the specific moral domains that the different foundations designate (Graham, et al., 2013).

Third, it is claimed that this *a priori* universal moral mind only constitutes the ‘first draft’ of every individual’s morality. During an individual’s life, the first draft is ‘edited’ through *cultural learning*. This means that the five domain-specific learning modules develop and create more specific modules within their moral domain as people grow up in a specific social and cultural environment. These specific modules constitute people’s more specific moral values, virtues and intuitions. For example, the innate fairness learning module develops several specific fairness modules during a person’s life that are culture-specific (e.g., a module for ‘not cutting in line’ in a culture in which waiting for your turn is an accepted means of allocating goods). To what extent an individual develops the different moral foundations, and the according sensitivities to domain-specific moral concepts and beliefs, into their actual adult morality, depends on the social and cultural environment one is brought up in and the individual’s personal experiences, especially during childhood. What specific moralities different people develop, in the sense of specific values and virtues, is therefore in itself not innately given. According to MFT, this process of cultural learning that is universally structured by the possibilities and limitations of an initial organization of the moral mind, can explain the stark differences as well as the (more fundamental) similarities that are found between moralities across persons, groups and cultures (Graham, et al., 2013, 2018; Haidt & Joseph, 2004, 2007).

Besides pluralist, nativist, and cultural learning elements, the fourth important component of the theory is its *moral intuitionism*. MFT builds on the Social-Intuitionist model of moral judgment developed by Haidt (2001), which claims that our moral judgments are directly *caused* by an intuitive process of moral evaluation. That is, an effortless and affective process that automatically results in a moral evaluation of like or dislike: ‘moral intuitions [are]. bits of mental structure that connect the perception of specific patterns in the social world to evaluations and emotions that are not fully controllable or revisable by the person who experiences them.’ (Haidt & Joseph, 2007, p. 381). People’s moralities are regarded as consisting of intuitive ‘input-output programming’ (p. 379), i.e., the more specific moral modules (e.g., ‘not-cutting-in-line’), which largely encapsulate moral judgment.

Deliberate reasoning is considered as *post-hoc* rationalization that does not have a direct influence on the already intuitively established moral belief or judgment. It only serves a social function in explaining the intuitively derived moral judgment to others, justifying what is already established and will not be altered. Haidt (2001) states that it is the kind of reasoning that is usually associated with a lawyer instead of a scientist, namely, fitting the reasoning towards an already accepted conclusion instead of impartial reasoning leading towards a yet unknown answer.

### Moral foundations questionnaire

To what extent an individual has developed the different moral foundations as part of his or her morality can be measured with the Moral Foundations Questionnaire (MFQ) (Graham, et al., 2011; MoralFoundations.org, 2013). The questionnaire consists of two parts. In the first, the so-called ‘relevance part’, respondents are asked to rate to what extent different general considerations are relevant to their thinking when judging between right and wrong (not at all relevant- extremely relevant). Each item taps into one of the moral foundations. For example, the item ‘Whether or not someone suffered emotionally’ is related to the care/harm foundation; the item ‘Whether or not someone acted unfairly’ to the fairness foundation; and the item ‘Whether or not someone conformed to the traditions of society’ connects to the authority/subversion foundation.

The second part of the survey, the so-called judgment part, asks about the respondents’ agreeableness with moral statements (strongly disagree-strongly agree). An example of a judgment-item related to the moral foundation of loyalty is ‘It is more important to be a team player than to express oneself’ and one related to purity/degradation is ‘Chastity is an important and valuable virtue’. Though the developers claim that the judgment part was designed to ask about context-specific moral judgments to complement the general moral considerations of part one, the judgment items rather consist of general and abstract moral principles, often applicable to a wide variety of contexts (Clifford, et al., 2015; Gray & Keeney, 2015).

The scores on the six items belonging to the same foundation are summed up, which results in an individual’s sum score per foundation. This expresses the degree to which a moral foundation underlies one’s moral beliefs and concerns (Graham, et al., 2011). The sum score for each foundation can consequently be used for testing association with other variables, such as attitudes and behaviours.

### MFT’s implicit concept of moral self

With the above description of the theory and the design of the questionnaire, it is possible to discern what concept of the moral self is implied by MFT. People’s moral identity is defined here by their endorsement of the moral foundations, expressed by their individual scores on MFQ. The moral self that seems to be implicitly supposed can then be characterized as a fairly stable moral personality or moral character. People’s intuitive moral regularities are developed by, and can be brought back to -or better: aggregated to- at least five general psychological moral dispositions that latently exist within the individual. As the development of these general psychological moral dispositions is measured outside of any specific context, it is assumed that these exist independently of any specific context and have a relatively stable hierarchy and efficacy across contexts. People’s general moral values are in this sense very much presented as moral personality traits. The moral self that underlies MFT seems to be an example of what Frimer and Walker (2008, p. 344) call an “essential” self-concept: a moral self that is ‘*unified, internally consistent and has an essence that exhibits agency across contexts*’. The essential nature of one’s moral self here consists of the endorsement or development of the general moral values, which is expressed by an individual’s MFQ-score. This does not mean that one’s moral values and moral self cannot further develop or change over time. Yet, it does suggest that this moral self amounts to a relatively stable moral character that causally affects decision making and behaviour in a similar way across different contexts and over a longer time frame. In its core, the essential aspect of this concept of moral self lies in the proposed intuitive moral regularities that produce a certain output when receiving a certain input and which can allegedly be summarized by abstract moral value scores without referring to any context.

### Leeway for a more dynamic concept of moral self within MFT

The described implicit conception of the moral self as a stable moral character is then derived from two interlinked sources. On the one hand, it follows from the main theoretical description of MFT, where domain-specific learning modules develop intuitive moral regularities that causally determine people’s moral judgments. On the other, it is derived from the nature of the questionnaire that asks about general and contextless moral principles. This presumes that these moralities can be effectively summarized into *general* moral values or virtues, functioning as individual moral traits and affecting the same kind of behaviour across contexts. This then sketches a quite rigid and determined picture of the moral self and the influence of people’s moral values on behaviour.

The view on the human being as having general (moral) traits determining behaviour across contexts, has been criticized by situationist theories that emphasize the influence of the situation and social context on (moral) decision-making and behaviour (e.g., Doris and Doris, 2002; Ross and Nisbett, 2011). Interestingly, Haidt and Joseph (2007) defend MFT against this possible critique and, thereby, create leeway for a moral self (without further developing it) that seems to be in tension with the concept of moral self that is suggested by the main theory and questionnaire. Here, MFT is placed in the tradition of virtue ethics and, while first describing people’s developed moralities as intuitions in the sense of input-output regularities, these moral modules are now also linked to virtues and to characteristics of virtues that suggest a more dynamic and contextually sensitive interpretation of people’s moral nature.

Haidt and Joseph (2007) state that virtues are characteristics of a person or traits, but not in the sense of broad behavioural dispositions or ‘global tendencies to act in a particular way (e.g., honest, brave) across widely varying circumstances’ (p. 386). Rather, virtues are described here as ‘dynamic patternings’, ‘capacities’, or situation-specific ‘social skills’ (p. 386): ‘[t]o possess a virtue is to have extended and refined one’s abilities to perceive morally-relevant information so that one is fully responsive to the local sociomoral context. To be kind, for example, is to have a perceptual sensitivity to certain features of situations, including those having to do with the well-being of others, and for one’s motivations to be appropriately shaped and affected.*’* (Haidt & Joseph, 2007, p. 386). Developing a virtue is a ‘comprehensive attunement to the world’ (p. 387) and, furthermore, it is explicated: ‘what it means for a personality characteristic to be a *virtue* and not simply a behavioural regularity, is largely that it consists in functioning well in a specific “sphere of existence.”’ (p. 387). Here, people’s moral character, consisting of certain virtues or moral values, receives a more dynamic and contextual nature in the sense that it attunes to and is embedded in the social context. This seems to presuppose a different relationship between the moral agent and his or her values, and, ultimately, a different concept of the moral self than the one that we derived from the main description of the theory and questionnaire.

Another aspect that the authors bring forward as relevant to MFT and that has been linked to virtue ethics is the role of narrativity in moral thinking and moral development. It is argued that through our moral intuitions, produced by our moral foundations, that narratives can become compelling moral stories. At the same time, it is through moral narrative that the intuitions of our moral foundations are socialized and developed into coherent moralities while growing up (Haidt & Joseph, 2007). The notion of narrativity is however neither further developed nor connected to a concept of the moral self. The connection with narrativity does seem to point towards a self that interprets and tries to make sense of oneself and its social environment, presuming a certain dynamic and context sensitivity as part of moral thinking.

The link that is made by MFT to virtue ethics and narrativity then opens the door to a more dynamic moral self that is sensitive to the specific situational context in which it finds itself. However, such a concept is not further developed, and, also, seems to be at odds with the implicit essential moral self that underlies MFT’s main theoretical elements and structure of the questionnaire. In fact, a more dynamic and contextual moral self with a corresponding relationship to one’s moral values, seems problematic for MFT’s idea that morality mainly consists of intuitive input-output regularities as well as for predicting judgments and behaviours on the basis of generally measured moral values. One of the main aims of the analysis in the following sections is to explicate and problematize this ambivalence in MFT and to propose a more viable concept of moral self for the empirical study of morality.

### Turning to phenomenology

To investigate and explicate the found ambivalence in MFT and evaluate its concept of moral self, we will now continue our analysis on the fundamental level of the moral self. As such, it seems prudent to turn to a field that has a long tradition in thinking about the (moral) self, namely *phenomenology*. Phenomenology is the philosophical field which systematically studies the first-person perspective of the experiencing and meaning giving subject. More particularly, in the next section, we turn to Ricoeur’s narrative concept of the self. This theory presents a thoroughly developed (moral) self-concept, where three central notions that have arisen from our above investigation form fundamental elements, namely: *moral character*, *a self that attunes to and is embedded in the social context*, and *narrativity*.

By explicating Ricoeur’s phenomenological concept of self we are, first of all, able to articulate a substantiated moral self that has fully developed the three above notions. Secondly, we are able to relate Ricoeur’s concept of moral self to the one that MFT implicitly assumes through its main theory and questionnaire. We can then evaluate the latter in terms of the former. This will also make clear what it would mean for MFT and its measurement tool if it in fact adopts a full-fledged dynamic concept of the moral self, like Ricoeur’s narrative moral self, and leaves its ambivalent position. Third, this brings us to suggestions as to how it may be possible to incorporate the found phenomenological insights into the empirical study of people’s morality.

## Ricoeur’s concept of the moral self

### Ricoeur’s phenomenological and hermeneutical approach

In this section and the next, we draw on the work of Paul Ricoeur, to present a thoroughly developed concept of the moral self which explicates ideas that MFT points to, but does not elaborate on. We will argue that this conception is ultimately at odds with aspects of MFT’s main theory and questionnaire.

Ricoeur’s overall aim is to develop a notion of moral selfhood that sails between the Scylla of the Cartesian essential Ego and the Charybdis of the Nietzschean splintered subject, offering an alternative to both extremes (Ricoeur, 1992, p. 1–16). His phenomenological approach consists of a hermeneutics of the self^5^ that seeks a position between these two alternatives. *Hermeneutics* can be understood as the philosophical approach that argues that the specific methodology of the humanities consists of interpreting (*Verstehen*) its objects of study, usually texts. Ricoeur applies this method of interpretation to the notion of self, as it is experienced by the first-person subject, to grasp and explicate this fundamental experience in a systematic way. Accordingly, we will call this hermeneutics of the self a phenomenological approach, since it stays true and further develops the first-person account.^6^ This hermeneutics proceeds by detours in order to tackle the question of identity or self by devising a theory of human action. Here it seeks to connect the questions “what” and “why” of action (what is action and how can we explain it?), which are the focus of analytic philosophy, to the question “who” (who is acting?), which is easily concealed but constantly presupposed by the first two. According to Ricoeur, it takes a hermeneutical approach that builds upon phenomenology, to bring forward this aspect of the acting person, which is selfhood. The only certainty this approach may claim is that of attestation. Attestation reaches an epistemic level that stands in opposition to the ‘ultimate and self-founding knowledge’ of the Cartesian Ego. However, it is not mere belief in the sense of *doxa*, which is inferior to knowledge. Rather, attestation links to the epistemic value notion of credence or trust and can ultimately be understood as ‘the assurance of being oneself acting and suffering’ (Ricoeur, 1992, p. 22).

### ***idem***-identity and ***ipse***-identity

Ricoeur’s concept of moral selfhood is a narrative notion of personal identity that should be understood as existing in time and as the ongoing dynamic interplay between two poles of identity: selfhood (*ipse*) and sameness (*idem*). In the following we will first describe these two distinct poles of personal identity and their specific way of existing in time. Subsequently, we will go into narrativity as mediating between them and constituting the moral self.

*Idem*-identity is identity in the sense of “sameness”. Overall, sameness is an answer to the question of identity in terms of “what?”, making re-identification possible. It has three different components: numerical identity, qualitative identity, and uninterrupted continuity. These three components may be contrasted with plurality, difference, and change, respectively. First, numerical identity means oneness in the sense that two occurrences of a thing are one and the same (I saw the plant in the room and now that I re-enter the room, I see the very same plant). Second, qualitative identity denotes the situation of extreme resemblance to the point of interchangeability. That is, between two things there is no qualitative difference (you are wearing the exact same dress as I!). Third, uninterrupted continuity harbours sameness as permanence in time, in the sense that one and the same individual goes through different stages of development (think of scrolling through someone’s photo-album or Facebook timeline covering several decades, and identifying the changing appearance as the same individual) (Ricoeur, 1992, p. 116–118).

Selfhood, or *ipse*-identity, is not sameness. It is another form of permanence in time, another way of answering the question of identity that is particularly relevant to the question of personal identity. While also things have *idem*-identity, *ipse*-identity belongs to persons only. Selfhood denotes reflexivity in the sense of a relation to self (Ricoeur, 1992, p. 1–2). It is a form of permanence in time that is an answer to the question “who?”, specifically when we are looking for the agent of an action: “who did that?” (Ricoeur, 1991). Persons are the privileged bearers of this notion of agency, in the sense that the actions belong to the self, the self owns them. So, the reflexivity resides in an agent being able to recognize herself as the subject of a certain action^7^. This self-ascription presupposes an identity –selfhood, self-designated by “I, myself”- that cannot be expressed in terms of sameness but that resides in one’s experience as a subject. The contrasting notion of selfhood is then not difference, but otherness.

When it comes to the permanence in time of persons, sameness (or *idem*) manifests itself as character. In other words, character points to one understanding of permanence in time, one way of answering the identity question: “who am I?”, which takes the form of the question “what am I?” as it is answered in terms of sameness or “what”. Character is seen as ‘the set of distinctive marks which permit the re-identification of a human individual as being the same’ (Ricoeur, 1992, p. 119). These lasting dispositions are related to habit, understood as a notion of sedimentation, and to acquired identifications with what is other than self, such as norms, values, or role models. Interpreted in this way, character offers stability, in terms of permitting the re-identification of persons, through the three senses of sameness: numerical identity, qualitative identity, and uninterrupted continuity. Though *idem* is emphasized at this pole of personhood, Ricoeur stresses that character is actually where *ipse* and *idem* overlap. Or better, ‘nearly overlap’, as their difference is not annulled. It is namely *my* character that belongs to *me*: ‘precisely as second nature, my character is me, myself, *ipse*’ (Ricoeur, 1992, p. 121). Or, as Ricoeur notes, character can be understood as the expression of selfhood in terms of sameness, i.e. ‘the “what” of the “who”’ (1992, p. 122).

Selfhood, on its own, harbours another form of permanence in time belonging to persons, namely self-constancy, in the sense of ‘that manner of conducting himself or herself so that others can *count on* that person. Because someone is counting on me, I am *accountable for* my actions before another. The term “responsibility” unites both meanings: “counting on” and “being accountable for”’ (Ricoeur, 1992, p. 165). The notion of keeping one’s word can be taken as emblematic for this self-constancy of selfhood. Indeed, the keeping of one’s promise appears ‘as a challenge to time, a denial of change: even if my desire were to change, even if I were to change my opinion or my inclination, “I will hold firm”’ (Ricoeur, 1992, p. 124). In this sense, for Ricoeur, the self has an inherently moral aspect from the outset. Keeping one’s word forms the opposite pole in Ricoeur’s model of permanence in time with regard to character, namely where sameness and selfhood are separated by an extreme gap (Ricoeur, 1992, p. 124). To make this pole of personal identity and its permanence in time more tangible, one can think of the practice of people who give each other their wedding vows in which they promise to take care of and be there for each other *no matter what* (whether it be a change of circumstances or of character). It is in this practice that we rely on the self and its self-constancy, independent of character.

To relate this back to the idea of *my* character, the pole of identity where *idem* and *ipse* nearly overlap, this notion of self-constancy or maintaining oneself is what characterizes my relation with my (moral) dispositions, such as the values I consider as my own. Ricoeur makes clear that this is a reflexive relationship, one of loyalty or fidelity towards these values and one of recognizing oneself in these values. This makes these values my own, while at the same time it permits a certain movement, flexibility, and adjustment in my relationship with values. It is exactly narrative that mediates this dialectic between myself and my values which is described in the next section.

### The moral self as a narrative notion of personal identity

As stated, Ricoeur’s concept of the moral self consists of the dynamic interplay of the two described poles of personal identity –*idem* and *ipse*, or character and self-constancy. This interplay is mediated by narrativity (Ricoeur, 1992, p. 140–151). Instead of what Haidt and Joseph (2007) emphasize, the role of narrativity is neither solely nor primarily pedagogical. Narrativity is, rather, constitutive for a viable account of the moral self. Indeed, because the (moral) self comes into existence by being narrated, the self should be understood as constructed by narrative (Halsema, 2019).

Before going into the technical details, it might be good to first get a basic idea of why Ricoeur gives narrativity a central place in his theory of identity. Think of the very first question you often get at a job interview: “Please tell us something about yourself.” In answering this question, you usually do not start enumerating your (best) character traits. Instead, you tell a story: the story of who you are. Naturally, through this story your character traits transpire. However, they are only one element in your story that encompasses your actions and behaviours and relations to others. If, at a later stage in the interview, you are asked which character traits make you perfect for the job, you might enumerate them. Yet, notice how this alone is seldom sufficient since you are usually asked to illustrate these with a concrete example. Here, again, your answer takes the form of a narrative: you tell the story of how flexibly you reacted when confronted with a sudden change in your schedule. The bottom line is that a full account of one’s identity takes the form of a narrative that mediates selfhood and sameness.

In his narrative account of the self, Ricoeur connects narrativity to the plot. The plot has an integrative function (Ricoeur, 1984, 1992). Understood as the movement of ‘discordant concordance’, the plot generates a ‘synthesis of the heterogeneous’ (Ricoeur, 1992, p. 141). More particularly, through the plot individual events and the story as a whole are configured, and brought into one coherent whole: ‘the narrative event is defined by its relation to the very operation of configuration; it participates in the unstable structure of discordant concordance characteristic of the plot itself. It is a source of discordance inasmuch as it springs up, and a source of concordance inasmuch as it allows the story to advance’ (Ricoeur, 1992, p. 142). The nature of the plot is therefore one of permanence and change.

As the plot makes a coherent whole of the heterogeneous elements in a story, it also provides the characters within the story with their identity. This is a narrative identity, correlating to the events of the story. A narrative, namely, does not describe events in an impersonal way. In a narrative, characters are linked to events as the ones who perform the actions or who are affected by the events, i.e., the narrative describes the character in its acting and suffering, defining the character. In other words, by telling the story through the structure of the plot, the questions of “who?”, “what?”, and “why?” are answered by connecting these answers through time. This gives the story a temporal configuration that makes it possible to follow it and, at the same time, renders the character a comprehensible identity that has duration in time (Ricoeur, 1984, 1992). As the character of a story achieves her narrative identity via the movement of the plot - mediating between change and permanence – this narrative identity itself also has the structure of the plot. As Ricoeur (1992, p. 143) notes: ‘characters, we will say, are themselves plots’. This means that the discordant concordance characteristic of the plot also applies to the character itself. This structure of change and permanence is the very structure of the dialectic between sameness and selfhood.

Now in the same vein, the identity of a person is constructed by telling one’s life story. In a hermeneutics of the self, it is the self that interprets her life through its acting and suffering and, simultaneously, her character through the mediation of narrativity with the structure of the plot. This interpretation takes the form of an appropriation, in the specific sense of making one’s own. Ricoeur (1992, p. 160–163) argues in this regard that narratives and life itself remain two distinct things. Think of how literary narratives cannot be simply applied to life but need to be appropriated: in this interaction between reader and text, the reader becomes the co-author of the meaning of a story. In the same vein, the self-narrative is an interpretation of one’s experiences of acting and suffering through an appropriation that organizes one’s life, integrating one’s past, present and future.

The mediation of narrativity, more precisely, relates the two poles of permanence in time of persons that Ricoeur distinguishes: self-constancy – the moral dimension of selfhood – and character. Through the narrative interpretation of the self, the self connects the question “who?” to that of “what?”. It provides the self with some ‘flesh on the bones’ through the story that is told and the dispositions of character that figure within it. At the same time, it gives the character the possibility to innovate when sedimentation has rendered traits rigid. It returns character to the movement that was lost in the acquired set of properties.

The constitution of one’s identity or the self through narrative, mediating *ipse* and *idem*, then provides character with a dynamic nature. This is, first of all, seen in the fact that, just as other stories, self-narratives can be told more than once and in different ways. Also, it is conceivable that different stories harbouring different kind of character traits apply to different social contexts (e.g., at work you are an authoritarian boss, but at home a timid husband). Furthermore, Ricoeur (1992) emphasizes that a person is only the co-author of her own life story as also other people tell and add to a person’s life story; entailing that one’s narrative identity remains open to changes and revisions until people stop talking about the person (Halsema, 2019). Finally, the dynamic nature of identity is shown in the interpretive act by which one configures the discordant events as part of a concordant life story. The appropriation of new events entails a constant reinterpretation of one’s life story and thereby of oneself. When having new experiences and figuring out what to do, these are interpreted in light of who you are, while attuning to the context of the specific situation. The appropriation of the experience as part of one’s life story then takes a mutual fitting by relating to and questioning oneself, as well as the specific social context. This implies a dynamic and context sensitive concept of the self, where acquired dispositions are brought back to the process of acquiring them, while self-constancy is given recognizable features. In this way the self is constituted as a permanence in time that is ever changing and developing.

### Narrative identity and moral identity

This narrative self remains faithful to the general claim of hermeneutics: it offers an interpretation of the self by a reconfiguration of cultural signs into symbolic circuits. This means that action and, accordingly, the self as interpreted in its acting and suffering, is always embedded within a certain culture and symbolically mediated. This entails that it takes place in a practical field that is articulated by rules, norms, values and signs (Ricoeur, 1984, p. 57–59). These give the practical field a meaning that is inherently public, i.e. available to and readable for actors within the field, while it also makes the interpretation of oneself and what should be done context-specific. Furthermore, this symbolic circuit or texture of action opens up to the idea of the prescriptive or normative, making it possible to evaluate action. Hence, neither action nor the self can be morally neutral.

In fact, narrative has had moral implications from its very start: right from the oral tradition of storytelling, which was about exchanging experiences and examples of exercising practical wisdom. In a similar way, fiction provides us with imaginative explorations of judging characters and actions. As Ricoeur (1992) makes clear, a narrative is never morally neutral, but peppered with evaluations. The plot, for instance, does not only logically structure a narrative, but also provides it with a certain end goal or good (implicitly) put on the horizon. Furthermore, narrative theory anticipates and supports moral theory by the concept of action that it offers (Ricoeur, 1992). This is a specific, layered notion of action, of which practices (basic actions, nesting relations, constitutive rules) and life plans (the narrative unity of a life as brought about by actual experience and fabulation) are important elements, forming an integrative whole expressing certain values and goals that are related to ‘the good life’. Narration, therefore, marks the transition between ascribing action to an agent and prescribing obligations to act to an agent. It is the narrative self that is the agent of moral action, for such notions as ‘the narrative unity of life’, ‘life plans’, ‘the good life’ assume both how life is rooted in biology and the way in which an agent regards this life as her own (Ricoeur, 1992, p. 178). This agent is a self-interpreting animal in the sense of Charles Taylor: interpreting and trying to bring into agreement her notion of the good life with her actions (Taylor, 1985).

At the same time, narrative identity, Ricoeur argues, is also characterized by the dialectic of the self and the *other* (Ricoeur, 1992). In this respect, narrative identity does not solely consider my own life and life plans, but it is also concerned with duties towards others and how to treat them well. The interconnectedness of narrative and moral identity entails, on the one hand, that narrating implies morally evaluating one’s actions, on the other hand, moral identity assumes that one is able to give a narrative account of one’s actions, reflecting upon them and giving reasons for them (Halsema, 2019).

Our interpretation of the self in its acting and suffering is thus always normative. This is already given by the necessarily interpretive approach that we take to our life and our experiences (Van Tongeren, 2020). These experiences are rooted in a way of being in the world that is always already normative, i.e. characterized by meaning and values. In this regard Sayer (2011) claims that we are ‘beings for whom things matter’, i.e. our relationship to the world is primarily evaluative, marked by import, significance, or, indeed, meaning. It is in these interpretations, or narratives, that our moral dispositions like virtues and values, defining our moral character, have their place and through which they are expressed. As explained, this entails a dynamic and context-sensitive relationship with moral values, which involves a constant relating and questioning of our values within a specific context to which one attunes. This leads to an idea of moral decision-making that amounts to moral evaluation in situation.^8^ This also means that, through the mediating role of the narrative moral self, the relation between our moral values and our behaviour is characterized by interpretation, making the effects of moral values on behaviour dynamic, in the sense of varying in kind and strength across contexts.

## Confronting MFT’s concept of moral self with Ricoeur’s narrative moral self

Let us retrace our steps. In the second section we explicated the moral self that MFT implicitly assumes through its main theory and questionnaire, while we also referred to three notions related to virtue ethics which the developers of the theory have linked to MFT: *moral character*, *a self that attunes to and is embedded in the social context*, and *narrativity.* Together, these three notions seem to point to an alternative, possibly more dynamic and context-sensitive moral self. However, such a moral self is not developed within MFT-scholarship. In Sect. 3, we turned to phenomenology to investigate what such a dynamic moral self would look like. Here we expounded on the narrative conception of the moral self, as developed by Ricoeur, to bring forward a thoroughly developed conception of the moral self that elaborates these three notions. We can now relate back to MFT to see what these insights can tell us about the moral self that it implicitly presumes through its main theory and questionnaire, i.e. how we can qualify MFT’s moral self in relation to Ricoeur’s narrative moral self, and, in its wake, what this suggests about empirically measuring morality.

### MFT defines the moral self solely in terms of ***idem***-identity

We ascertained in Sect. 2 that MFT’s implicit conception of the moral self consists of an internalization of general moral values or development of general moral virtues that function as character traits and together form a fairly stable moral personality. This was derived from the notion that MFQ measures the extent to which distinct general learning modules (the moral foundations) are reflected in people’s developed moralities (consisting of intuitive input-output regularities). The questionnaire consists of general items such as: ‘When you decide whether something is right or wrong, to what extent are the following considerations relevant to your thinking? –‘Whether or not someone suffered emotionally’/ ‘Whether or not someone acted unfairly’ etc., each tapping into one of the five defined moral foundations. The aggregate measure of each foundation can be regarded as the endorsement of a *general* moral value or virtue. These are subsequently used to explain different kinds of attitudes and behaviours across contexts. As the general moral values are measured outside of any specific context, it is assumed that their endorsement exists independently of any specific context and that they have a relatively stable hierarchy and efficacy across contexts. Together with the idea that these general moral values are the aggregate reflection of input-output moral regularities, this amounts to an essentialist concept of moral self, where people’s general moral values or virtues can be regarded as dispositional moral traits that, through moral regularities, intuitively and causally determine certain attitudes and behaviour.

Following Ricoeur’s terminology, the above description of MFT amounts to a conception of the self that -at most- can be considered in terms of *idem*-identity. People’s score on the MFQ is an answer to the question “who am I (morally speaking)?” in terms of *what*: the moral values that someone supposedly endorses in general. It is this general moral value endorsement that defines the person morally and that bears the characteristic of sameness. It constitutes a moral character that offers stability through the three senses of sameness: numerical identity, qualitative identity and uninterrupted continuity.

Importantly, this is not to say that MFT’s implicit moral self can be equated to Ricoeur’s concept of *idem*-identity or to what he brings forward as the *idem*-aspect of character. Where the moral regularities may come close to what Ricoeur calls “habits”, the innate origin of MFT’s moral character is something Ricoeur would reject (e.g., Changeux and Ricoeur, 2000). By interpreting MFT’s implicit concept of moral self in terms of *idem*-identity we merely emphasize its permeation with sameness.^9^ It is an example of what Ricoeur calls ‘…the inscription of character in Sameness’ (Ricoeur, 1992, p. 119 nt. 4). That is, an idea of moral self or moral character that is simplified and ossified as it earns stability solely in terms of “what” without making reference to a “who”, nor to the reflexivity of self-constancy. In other words, what is hard to grasp for MFT is the idea that *someone is relating to one’s own moral values*. For Ricoeur, in contrast, it is clear that even at the pole of moral character the self never vanishes entirely, as ‘one cannot think the *idem* of the person through without considering the *ipse*, even when one entirely covers over the other’ (Ricoeur, 1992, p. 121)

Without doubt, the three senses of sameness are vital to empirical analyses: first, scores on the different moral values belong to one and the same (numerical) individual, defining and individualizing him or her in moral terms, giving the opportunity of re-identification. Second, MFQ-scores also offer stability in the sense of qualitative identity. Two individuals that have the same score on a certain item or on the aggregated foundation score are regarded as the same, in the sense of being similar. It gives the ability to compare individuals in moral terms. Thirdly, the MFQ-score defining an individual’s moral character offers stability in the sense of uninterrupted continuity. It offers continuity of an individual in moral terms over time and in different situations.

All three senses of sameness, harboured by an individual’s moral character in terms of MFQ-scores, are prerequisites to doing meaningful empirical analyses. It is because scores can be attributed to one and the same numerical individual that certain combinations of scores can lead to associations between variables (such as values, attitudes, and behaviours) on the population level (e.g., regularly finding the combination of relatively high scores on variable X and Y within different individuals, while also regularly finding the combination of relatively low scores on X and Y, leads to a positive association between the two variables). To establish such associations we need, of course, a measure of qualitative sameness between individuals. These associations can only be meaningful when the scores express a certain continuity in an individual’s morality.

As should be clear, though essential to empirical psychological investigation, this approach easily loses sight of the other crucial aspect of moral personhood that has been brought forward by Ricoeur: selfhood. This is, of course, not a particularly surprising conclusion, nor a fault of psychological methods. The psychological sciences necessarily take an observational and thus third-person perspective, aiming at scientific objectivity. However, this does not make the first-person perspective, which phenomenology can bring forward, less relevant for understanding morality and moral behaviour and thus for the central aims of (moral) psychology. Phenomenology is able to articulate the person as subject and its structures of experience that are central to the moral life, reaching a verity level of attestation. Empirical psychology is able to objectify these experiences, losing a certain richness in experiential information, but enabling methods of generalization on the population level and of prediction that can claim scientific objectivity. The approaches complement each other.

At the same time, following our analysis, it can be concluded that the three notions of virtue theory that have been related to MFT actually presuppose *ipse*. When general moral value theories would accept such a conception of the moral self, as we think they should, this is not without implication for their theory and measuring methods. In the discussion we will further go into what it would mean for the empirical study of morality to incorporate *ipse*-identity to a certain extent.

### Reflexivity (***ipse***) in a theme park

We will now continue by making clear what a moral self in terms of *idem* without *ipse* and the mediation of narrativity amounts to and how it contrasts to a moral self that does incorporate them. This is best described using an example. Let’s take the situation where you are waiting in line for a ride in a theme park. Two boys of about 10 years old sneak in and cut in line just in front of you. Now say that you in general highly endorse the moral value of fairness in the sense of MFT. You have developed a certain sensitivity for social situations in which this value is jeopardized, as in the situation you find yourself in right now. In fact, part of the development of the fairness foundation into your morality is the development of the more specific not cutting in line rule. The not cutting in line rule is what you, in general, find morally important. In case you would fill in the MFQ you would score highest on the fairness foundation, your score on the care foundation would be somewhat lower and the binding foundations again a bit lower. If the idea of a moral self solely in term of *idem* is correct then this situation would always lead you to judge the behaviour by the boys as wrong (following your intuitive dislike evaluation) and, accordingly, to consider telling them off (or some other proportional action) as the right thing to do.

Of course, such a course of events is conceivable. But another scenario may just be as conceivable. Namely, that you, though perhaps initially put off by the cutting in line of the boys, remember yourself at that age. How you used to play around with your brother, being cheeky sometimes but not intending any harm. Feelings of affection and care come up and, at the same time, you can feel again the emotional stress when some older person told you off in these cases, as well as the disappointment you imagine the boys would feel when you tell them to get out of the line. You look around you, there are mainly adults in the line. Besides, it’s a really quiet day at the theme park and the waiting times for the different attractions have been short. You decide to let them be and have them enjoy their ride. Now the point here is not that this scenario is more probable than the former, or that this is necessarily the right decision. Rather, the point is that it is at least imaginable that someone would reason and feel like this, even when fairness is regarded as his most important value in general, and that it seems to be a possible morally acceptable way of dealing with the situation. In fact, it is conceivable that, to the decision maker, this decision is what it means to act “fair” in these circumstances.

Would such a scenario be possible if the moral self solely consists of *idem*-identity? It seems not. What the second story implies is *reflexivity*. Recall how reflexivity refers to the relationship towards oneself, i.e. an agent is able to recognize herself as the subject of a certain action. The question becomes what your *own* values tell you to decide in this situation. In other words, what is emphasized in terms of selfhood is not that fairness, care and binding values are the ones that are always called upon by you when you take moral decisions. Rather, what is important is that in recognizing values like fairness, care and loyalty as your own values when making a moral decision in a situation, you recognize yourself as a moral agent. 10 That is, these values are part and parcel of your moral identity, or selfhood. The permanence in time at play here is self-constancy: you are responsible for your actions in the double sense of others being able to count on you and you being accountable for your actions. You are holding firm, not in the sense that you stubbornly hold on to fairness (‘whoever comes in line first, is first to take the ride’) but that you recognize yourself in your decisions and actions. Letting the boys get in front of you in this situation is consistent with who you are.

Note how the decision here is highly influenced by the concrete social context in which you find yourself. The moral self is embedded in a concrete practical field of actions that comes with its own specific rules and values to which it attunes. You are in a theme park, i.e. a place imagined and designed with primarily children and young adults in mind. The park, from the attractions to the food options and from the availability of baby change rooms to the walking routes, is catered to their desires and needs. Adults are ‘less important’ in such a place. It’s even the implicit rule of theme parks to be in a good mood, have fun and let children do things that they normally can’t do, skipping the line may be one of these things. You pick up the signs that help you guide your moral decision in this specific situation. Before letting the boys go first, you checked whether there were no other children waiting in line. You estimated that the other adults in line will not mind that you let the boys go first. Furthermore, lecturing the boys about the importance of queuing seems especially “out of place” and even against the unwritten rules of a theme park. Letting the boys go first is a decision that attests to the moral self as embedded in and attuned to a concrete situation.

Given the above analysis, we argue that whenever you need to take a moral decision the narrative plays a mediating role between yourself and your values. Before taking the decision, you were reminded of you and your brother at the same age, a concrete episode in your life story. How the fairness, care and binding foundations play out as moral values that may be called upon to guide moral decisions in concrete situations was influenced by this. As we have discussed above, Ricoeur argues that narrative mediates the character traits of *idem* and the self-constancy of *ipse*. Fairness, care and binding values are taken up in a concrete situation by narratively connecting them to the moral agent. In appropriating these values, you make them your own, you acknowledge them as part of your self-narrative, or life story. This process of appropriation is done through a plot; integrating the heterogeneous through a ‘discordant concordance’, providing a narrative unity to different events and situations over time and figuring the values that are important to you. The narrative answers the question *who* is the moral agent? In answering this question, you tell your life story, i.e. you relate to those aspects of your life story, how you acted and were affected, that are important to this situation and that are imbued with evaluations.

Here, in retelling your life and connecting it to you as a kid, having fun with your sibling, not being preoccupied with rules and conventions but without intending any harm, you answer the question of the *who*. At the same time, your values are given life by being confronted with the narrative of the moral agent in a concrete situation. In finding yourself in line in a theme park with the boys trying to get in front of you, you are confronted with the question what these values mean for you in this specific situation. In answering that question, you make use of narrative to reinterpret fairness, care and binding values, ultimately relating them to who you are. In a theme park confronted with boys who want to get in front of you in line, you tell of yourself as a person who values fairness, care and binding values and has a brother with whom he played and had fun. Subsequently, the decision you take is informed by and shapes the story you tell about yourself. The self-narrative organizes your life, integrating your past, present and future, and you bear this life story with you and put it at play in every moral decision you take. Letting the boys go first is a decision that attests to the narrative nature of the moral self.

### Problems for predicting behaviour based on general moral values

Now, what does the above analysis mean for predicting behaviour from general moral value measures? First of all, it can be said that general moral questionnaires, like MFQ, do tap into people’s self-narrative. Questions that ask you to reflect on certain moral considerations and moral statements ask for self-interpretation and actually presuppose *ipse*-identity. Questionnaires measuring moral values or moral personalities, then, do not go beyond, but rather make use of people’s self-narratives. The result is a certain reflection of (parts of) this self-narrative. The main problem arises with how this reflection is subsequently interpreted and treated. Reduced to a stable set of general moral value traits, the self-narrative loses its defining features, namely that it is an interpretation that is in need of constant reinterpretation; an idea of self that is in constant flux and fits and adjusts itself to the different contexts it encounters. With this reduction, one’s moral identity loses its defining *ipse*-aspect, exposing at least two problems for the prediction of behaviour from general measures of moral values.

The first is that a general questionnaire fails to grasp the flexibility of decision-making and action that our values permit, due to the reflexivity given in *ipse*-identity. Though MFQ-scores may be regarded as a possible expression of one’s interpretation of the moral self in terms of general moral values, it fails to grasp that these values are related to and reinterpreted in every new situation through the mediation of narrative, in order to decide what to do. By fitting the episode of the decision situation into the other episodes that make up our life story, we again question and appropriate our general moral values in a way that is specific to the decision’s context and that fits them into who we are. Furthermore, within a specific situation, you may call upon specific parts or episodes of your self-narrative. In other words, a particular version of you that does not feature as prominent in the general interpretation of yourself (e.g., think of recognizing yourself in two little kids in a theme park). The meaning that general moral values acquire within a specific decision situation, their mutual relationship, and what kind of behaviour they determine, is thereby highly contextual and hard to predict. This flexibility is not simply incorporated by measuring values (or modules) that are somewhat more specific or contextualized (like the cutting in line rule), as a person still needs to relate to such (more specific) principles within a particular situation, implying the *ipse*-aspect of the moral self. Though measuring more specific values can be expected to render somewhat better results, as it may hold better information of the role of certain specific moral values in that context.

A second problem that the above analysis suggests is that individuals may have dispersing understandings of the moral concepts figuring in the questionnaire. If we, indeed, understand our moral self and moral values through narrative, this suggests that when we fill in a questionnaire, which triggers reflection on moral concepts and values, we also use certain episodes and situations to see what we think. These episodes can be ones that we have appropriated as part of our life story. This means that broad moral concepts like “unfair treatment” or “emotional suffering” are understood through and are given meaning within our particular life stories. The meaning we give is thereby never really general, but always has some specificity. This can have the result that, for instance, unfair treatment for one individual is predominantly understood in terms of the unequal treatment of different groups of people by institutions, due to one’s life story, while another person may predominantly relate to other kind of episodes, such as about persons that give themselves a preferential treatment (i.e., when cutting in line or when cheating in a game). People may also think of widely differing contexts, ranging from unfair treatment in the work-place to the unfair treatment of animals. The problem is that these different understandings are not reflected by people’s scores on generally formulated questions. Behind two relatively high scores on the importance of fairness for one’s moral judgments can exist two quite different meanings, stemming from different narratives and life stories. As it is assumed that these are the meanings behind the scores that trigger behaviour, it seems logical that this dispersion affects the predictive value of these items. Making items more specific to a certain context may be a way to reduce this problem. This is of course at cost of the idea of a general moral value that has predictive value over many different contexts. But, as we have argued, this is not how we believe that the moral self in relation with its moral values functions.

## Discussion

Like any full-fledged moral theory, either normative or descriptive, MFT assumes a conception of moral self. Using insights from Ricoeur’s notion of personal identity, we have argued that MFT assumes an underdeveloped concept of moral self, which is reflected in a naïve way of measuring people’s morality. Following Ricoeur, we have presented an improved concept of moral self for the empirical study of morality.

As we have aimed to make clear, Ricoeur’s narrative concept of the moral self connects to aspects of virtue theory that the developers of MFT themselves consider as part of their theory. The notions of *moral character*, *a self that attunes to and is embedded in the social context*, and *narrativity*, are elements that are claimed by Haidt and Joseph (2007) to underlie MFT, but which are neither further developed nor connected to a more elaborated concept of moral self. With Ricoeur’s notion of the moral self, we were able to further develop these elements and show what it would mean for MFT and for measuring morality when these would be seriously incorporated. Our analysis thereby exposes an ambivalence within MFT on a fundamental level. Accepting the proposed more substantial moral self leads to a clash with those elements of the core theory that may only claim a moral self in terms of *idem* and to rejecting its according method of measurement (MFQ).

In particular, considering MFT’s theory, it follows from our analysis that accepting a certain attunement to the social context and a place for narrativity in moral judgment and decision making is not intelligible without accepting the reflexivity of the self (*ipse*). That is, if MFT is serious about incorporating these elements, it must acknowledge a moral self who relates to one’s moral values and moral regularities, if only, by interpreting them and deciding between conflicting or multiple possible ones in a situation. Yet, this, in turn, strikes at the roots of MFT’s core theoretical idea of morality simply consisting of input-output moral regularities that can be aggregated in terms of general moral dispositions and the implicit essential concept of moral self that follows from it. MFT would have to leave its underlying idea of decision-making where a defined set of general moral values as moral dispositions causally determine behaviour across contexts. Instead, general moral values should rather be regarded as touchstones that are called upon in decision-making to figure out whether a specific action is in line with one’s moral understanding of oneself. Ricoeur’s concept of the narrative moral self makes such an idea of decision-making and its according interpretive and dynamic relationship with one’s values intelligible. It is the narrative aspect of the moral self that leads the way here, where action depends on one’s ideas about ‘the good life’, turning decision making and action into an interpretation of the self in situation, offering flexibility as well as stability.

In its wake, the improved conception of the moral self, incorporating both *ipse*- and *idem*-identity and the mediation of narrativity, exposes considerable difficulties for measuring people’s morality in general terms and using these to predict behaviour across a variety of contexts. Our analysis implies that the specific meanings that our moral values receive, their importance vis-à-vis each other, and the decisions and actions they determine are situation-specific and, therefore, cannot simply be measured in a general way, out of context. Another aspect complicating prediction from general measures of moral values, is that the concepts used in the questionnaire do not have a univocal interpretation. Following our analysis, people give meaning to moral concepts, such as ‘fairness’ or ‘emotional suffering’, through their particular life stories. Similar scores on a moral foundation may therefore actually harbour quite diverting meanings.

In sum, due to the mediation of the narrative moral self, the relation with our general moral values and –thereby the relationship between general moral values and behaviour- is interpretive in nature, instead of being characterized by causal determination. Therefore, the influence of moral values on behaviour varies in kind (i.e. depending on its specific meaning) and strength across persons and contexts. This dynamic relationship makes it fruitless to predict behaviour from moral values when *not* taking these specificities into account, as empirical moral value theories try to do.

As discussed, the fact that MFT’s measurements solely reflect *idem*-identity, seems to be part and parcel of the observational, third-person stance of the empirical sciences. By definition, this view focuses on the “what” of the “who”. For empirical sciences, the phenomenological structure of the self is hard to grasp. On the other hand, empirical psychology is able to make generalizations about populations and predictions and can give insight into broader tendencies, which are not part of the phenomenological toolbox. It is therefore not a matter of choosing one or the other, but rather of finding ways where the two realms can complement each other.

Though *ipse*-identity may be hard to grasp directly by empirical investigation, it can, to some extent, be taken into account in the used measuring method. In the following, we suggest possible ways for the empirical investigation of morality to incorporate the explicated phenomenological insights on the moral self. With regard to empirical moral value theories, this entails a substantial revision of their measuring method. First of all, in light of the dynamic and context-sensitive relation with our moral values, studies focusing on the influence of morality on moral behaviour should limit their study to a delineated context (i.e., a certain professional environment, a school, a retirement home, car drivers, or the playground). As people’s moral values get their specific meaning and importance within a decision context, abstracting from this will inevitably lead to a loss of information. Though a certain level of generality is of course necessary to make general empirical claims, the more abstract these get, the more meaningless they become. For a better understanding and prediction of moral behaviour it is, therefore, necessary to understand the particular meanings of moral values and the importance given to them by individuals within that context.

This brings us to a second implication of our analysis for the study of morality in relation to behaviour. Given the context specificity of our moral considerations, as well as the richness of our self-narratives in terms of moral meaning, studies should take an exploratory rather than a confirmatory approach to measuring morally relevant phenomena, like moral values. Instead of imposing and limiting the choices of what can be possibly considered morally important or relevant for people from the top down, studies should start from a bottom-up approach to map out what morally matters to people when deciding within a certain context. This could be done by having quantitative studies be preceded by more qualitative investigations in which people’s moral considerations are brought forward in a narrative form.

Here, the empirical study of morality could learn from qualitative approaches in the behavioural sciences. For instance, discourse analysis and social practice theory map out people’s considerations, interpretations and social practices, playing a role in decision-making in a specific behavioural context (see Dickinson et al. (2010) for an interesting study on people’s considerations with regard to climate change and their choice on holiday travel mode). After mapping out such context-specific interpretations and considerations, these can subsequently be quantified into context-specific measures. Such a procedure is a double-edged sword, as it reveals the specific moral considerations that play a role within a certain context and, at the same time, measuring more precise concerns harbours less risk to arrive at diverting meanings. Also, the measurement of importance of such context-specific measures vis à vis each other can be expected to be more robust. Another procedure in which a qualitative phase informs quantitative analysis has been conducted by Boyd et al. (2015). In this study, on the influence of general moral values on everyday behaviour, participants were asked to describe their most important values in relation to who they are in their own words. From these narratives, people’s values were deducted by counting certain theme words. These measurements proved to be more successful in predicting behaviour than pre-established moral scales. This indicates that starting from such narratives is a better way to grasp people’s moral identities. Following our analysis, applying such a method to a more delineated context of behaviour may prove to enhance predictions further.

A third point of enhancement, following from our analysis, is designing the method of measurement in such a way that respondents actually need to rank moral concerns against each other. The dynamical aspect of the moral self, by relating to and interpreting one’s moral values within a decision situation, involves weighing conflicting concerns against each other. This is not reflected in a general questionnaire, such as MFQ, where all items can receive the same score.

An example of a methodology that has been thoroughly developed in the last decades and which incorporates a large part of the above suggestions is Q-methodology. Combining qualitative and quantitative methods and focusing on one specific context, Q-methodology seems a promising approach for the empirical study of morality (Brown, 1980). Here, different perceptions about a subject, existing within a population, are measured. The first step is to exploratively collect statements, opinions, preferences etc. about a certain subject. For instance, this is done through conducting interviews with focus groups. Then, this broad collection is brought back to a representative set of statements. Subsequently, participants rank these statements vis à vis each other in terms of agreement. These individual views are correlated, resulting in several different perceptions on a subject that are, to a certain extent, generalizable to the population (Brown, 1993). Such subjectivities seem more insightful to understanding behaviour and the role of morality within a practical context and may also prove to be better predictors of behaviour within that context than general moral measures, which turn narratives into a general moral disposition. Of course, all above suggestions for enhancing the measurement of morality and its prediction of behaviour is subject to further empirical study.

Indeed, the described quantitative methods can still only grasp persons in terms of *idem*. However, they do this while taking into account the dynamic aspects and context sensitivity of *ipse*-identity, to some extent. With regard to empirical moral value theories, and MFT in particular, the question is to what extent these are willing and able to incorporate this aspect of personhood in their theory and method. In this article, we have aimed to show that if theories, like MFT, are serious about incorporating a more developed concept of the moral self, like the one we have explicated in this paper, and we believe they should be, their core theory as well as their method of measuring people’s morality needs substantial revision.

## Data Availability

Not applicable.

## Footnotes

1 Note that Schwartz Theory of Basic values entails a broader set of general values than just morally relevant ones.

2 A similar problem has been identified with regard to the association between people’s moral judgments and their behaviour, known as ‘the judgment-action gap’ (see e.g., Lapsley and Narvaez (2004). In this paper we will not go further into this discussion as we confine ourselves explicitly to empirical moral value theories and the relation between moral values and behaviour. However, we do not rule out that findings and insights in our analysis may prove relevant to that discussion.

3 The developers state that their theory is primarily about moral virtues instead of moral values, given the cultural developmental element that is part of it. Furthermore, it is stated that the moral foundations themselves are not one’s moral virtues, they are the fundamental learning modules that give rise to people’s broader set of culturally embedded virtues (see below) (Graham, et al., 2009). However, in the broader literature on MFT, the moral foundations themselves are often referred to as people’s (basic or general) moral values, including in studies by its developers. Furthermore, the questionnaire itself does not contain anything that designs it specifically for tapping into moral virtues instead of moral values. The items in the questionnaire seem to be suitable for both. We therefore refer to what is measured by MFQ as one’s moral foundations, general moral values, or general virtues interchangeably.

4 The first two are considered the ‘individualizing’ foundations, as these protect the interests of the individual. The last three are considered the ‘binding’ foundations as they protect the interests of the group (Graham, et al., 2009). Note that the authors state that they are confident about the existence of these five moral foundations but that more may be discovered. See Graham et al. (2013) for a checklist noting the criteria candidate foundations should satisfy before they can be recognized as a genuine one. Promising candidates that have been proposed are the values of ‘liberty/ oppression’, ‘equity/ undeservingness’, and ‘honesty/ lying’ (Graham, et al., 2018).

5 We are aware of the fact that Ricoeur’s notion of a narrative self has not been uncontested. In particular, we are familiar with the critique of Zahavi (2007, 2008 and, 2014), who argues that a phenomenological, embodied notion of selfhood precedes and underlies the hermeneutical, narrative self. A full treatment of this issue goes beyond the limits of this article. Nevertheless, we do want to stress that we are of the opinion that within Ricoeur (1992), and in particular when read against the background of other parts of his oeuvre, phenomenological and hermeneutical considerations are combined, leading to a notion of selfhood that is both embodied and discursive. Accordingly, we make no strict distinction between phenomenology and hermeneutics in this article. For an in-depth defence of this interpretation of Ricoeur’s notion of selfhood, see Halsema (2019).

6 Ricoeur clarifies his position in Ricoeur (1975). He rejects one specific version of phenomenology; the idealistic one of the early Husserl. Apart from that, he sees a relationship of ‘mutual belonging’ between phenomenology and hermeneutics. On the one hand, hermeneutics builds on phenomenology as its necessary presupposition. On the other hand, the goal of phenomenology – the return to the *Lebenswelt* – cannot be attained without the hermeneutical presupposition of interpretation.

7 In the same sense this reflexivity of the self is contained in recognizing one’s body as one’s own body, i.e., in Husserl’s idiom *Leib*; or in one’s thoughts as one’s own thoughts, i.e., as belonging to oneself.

8 Ricoeur discusses the moral evaluation in situation under the heading of the Aristotelean concept of *phronesis*, see especially study 7 and 9 of Oneself as Another (1992). While an extensive discussion of *phronesis* in the work of Ricoeur falls outside of the scope of this paper, it is noteworthy that he explicates the relationship between Aristotelian ethics and Kantian morality in Ricoeur (2007). The Kantian framework is crucial in delimitating ‘the hard core of the issue’, that is designating morality as the realm of the obligatory (Ricoeur 2007, p. 45). Aristotelian ethics situates moral decision-making in concrete situations against the horizon of a person’s ideas about ‘the good life’. It is here that virtues, understood as ‘excellences of action’, take central stage: ‘Each one of these excellences picks out its intended good against the background of an open-ended intention magnificently designated by the expression of a “good life” or, better, “living well.” This open horizon is inhabited by our life projects, our anticipations of happiness, our utopias, in short by all the changing figures of what we take as signs of a fulfilled life.’ (Ricoeur 2007, p. 50). The notion of *phronesis* enters the picture because the exercise of virtue in concrete situations cannot be separated ‘from the personal quality of the wise human being – the *phronimos* – the sensible person.’ (Ricoeur 2007, p. 54).

9 It is in this specific sense that we will refer to MFT’s concept of the moral self in terms of *idem* in the rest of our analysis.

10 This decision process does not necessarily have to take a conscious deliberative form, but could also be intuitive.
